# Drug-Loaded Polymeric Nanoparticles for Cancer Stem Cell Targeting

**DOI:** 10.3389/fphar.2017.00051

**Published:** 2017-02-14

**Authors:** Binbin Li, Qinghua Li, Jingxin Mo, Honglian Dai

**Affiliations:** ^1^State Key Laboratory of Advanced Technology for Materials Synthesis and Processing, Wuhan University of TechnologyWuhan, China; ^2^Biomedical Materials and Engineering Research Center of Hubei ProvinceWuhan, China; ^3^Department of Neurology, Affiliated Hospital of Guilin Medical UniversityGuilin, China; ^4^Key Laboratory for Stem Cells and Tissue Engineering (Sun Yat-sen University), Ministry of EducationGuangzhou, China; ^5^Department of Histology and Embryology, Zhongshan School of Medicine, Sun Yat-sen UniversityGuangzhou, China

**Keywords:** cancer stem cell, drug-loaded, polymeric nanoparticle, cancer therapy

## Abstract

Cancer stem cells (CSCs) have been reported to play critical roles in tumor initiation, propagation, and regeneration of cancer. Nano-size vehicles are employed to deliver drugs to target the CSCs for cancer therapy. Polymeric nanoparticles have been considered as the most efficient vehicles for drug delivery due to their excellent pharmacokinetic properties. The CSCs specific antibodies or ligands can be conjugated onto the surface or interior of nanoparticles to successfully target and finally eliminate CSCs. In this review, we focus on the approaches of polymeric nanoparticles design for loading drug, and their potential application for CSCs targeting in cancer therapy.

## Introduction

Cancer is widely viewed as the major reason of death in the world (El-Serag, 2001). Although decades of efforts of research and billions of funding have enhanced our understanding of the underlying mechanisms of tumorigenesis, cancer caused mortality remains running at a high level nowadays (Oishi and Wang, 2011). Recent studies have suggested that, the growth of tumors is similar to that of normal proliferative tissues, fueled by limited numbers of dedicated stem cells that are capable of self-renewal, called cancer stem cells (CSCs) (Reya et al., 2001; Clarke et al., 2006; Adams and Strasser, 2008; Dick, 2009; Wilson et al., 2009; He et al., 2016).

The concept of CSCs was introduced firstly in Bonnet and Dick (1997). CSCs have been identified in multiple malignancies, which are capable of self-renewal, and drive the propagation and development of a tumor (Jordan et al., 2006; Rosen and Jordan, 2009; O’Brien et al., 2010; Clevers, 2011). Due to these crucial roles, failure to efficiently eliminate CSCs during conventional therapy may lead to tumor relapse and metastases. Thus, CSCs are considered as a critical issue to be addressed in the cancer therapy (Dean et al., 2005; Li et al., 2015). Quite a lot of efforts have been put into the study of anti-CSCs strategies to completely eliminate the CSCs population, and some approaches have gained encouraging therapeutic outcomes, such as inhibiting the signaling pathways related to self-renewal to interfere the CSCs proliferation and tumor growth, breaking the tumor microenvironment to decrease the interaction between CSCs and cytokines, and targeting the markers on CSCs surface to locate and destroy CSCs etc. (Beck and Blanpain, 2013; Chen et al., 2013; Lu et al., 2016). Within these strategies, the hottest research in the field of cancer therapy is CSCs targeting, because of its ability to improve the survival of cancer patients, especially patients with drug resistance in recent years (Zhou et al., 2009).

However, the sensitivity of the drug changed usually in the tumor due to either genetic or epigenetic alterations (Zahreddine and Borden, 2013). This would lead to acquired resistance following the change of gene expression patterns, and eventually drug selection during cancer treatment. Drug resistance results in a series of problems including: the increase of CSCs population, disease relapse and tumor metastasis during the period of cancer treatment (Zhou et al., 2009; Takebe et al., 2011).

A large number of efforts have been done to minimize the harmful impacts of drugs during the process of cancer therapy (Vinogradov and Wei, 2012): (a) preventing the side effects on the nearby cells and tissues; (b) increasing the drug accumulation and efficacy in the lesion; (c) developing novel drug delivery and targeting systems.

The key factor to solve these issues above is to allow the CSCs exposed to the high enough drug concentration in cellular environment. Numerous novel drug delivery strategies have been developed with the rapid advances of nanotechnology. The nanosized drug delivery system can be designed to conquer the known disadvantages of the anticancer drugs, for instance, the low bioavailability and high cytotoxic side effects (Gupta et al., 2009; Kim et al., 2010). In one case, a novel nanogel-drug conjugates based on membranotropic cholesteryl-HA (CHA) has been synthesized for efficient targeting and suppression of drug-resistant tumors. These conjugates could significantly increase the bioavailability of poorly soluble drugs with previously reported activity against CSC, such as etoposide, salinomycin, and curcumin. The resultant CHA-drug nanogels demonstrated higher cytotoxicity in CD44-expressing drug-resistant human breast and pancreatic adenocarcinoma cells compared to that of free drugs and non-modified HA-drug conjugates. Approaches using polymeric nanoparticles to encapsulate drugs for CSC targeting attract more attentions in cancer treatment because of the advantages including the controllable release of drugs, structural design for targeting, and functional design for diagnosis (Wei et al., 2013).

In this review, we first introduce the specific characteristics of CSCs and the common strategies for CSCs targeting. We then outline the current polymeric nanoparticles used for CSCs targeting in the application of cancer therapy.

## Current Cancer Therapy via CSCs Inhibition

**FIGURE 1:**
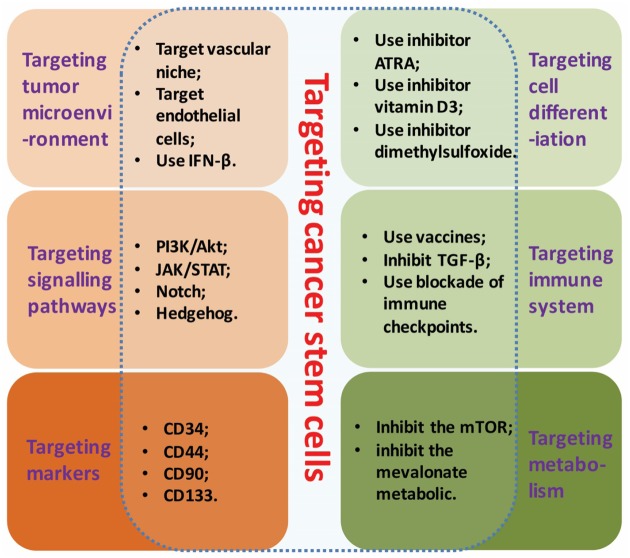
**Strategies of CSCs targeting.** Strategies of CSCs targeting based on its specific molecular properties include: interfering the growth microenvironment, targeting the biomarkers, inhibiting the key signaling pathways, targeting cell differentiation, targeting immune system and targeting metabolism.

With the recent exploration advancing in CSCs, the CSCs targeted therapy has brought a new hope to the cancer patients. The identification and characterization of CSCs have revealed numerous strategies of cancer treatment in-depth via specific molecular therapies (Visvader and Lindeman, 2008): for example, interfering the cell growth microenvironment of a tumor; targeting the specific biomarkers of CSCs; or inhibiting the key signaling pathways to interfere CSCs activity. Strategies of CSCs targeting based on their properties were showed in **Figure 1**.

### Targeting the Tumor Microenvironment

A niche is the CSCs intrinsic property built by tumor microenvironment to prevent from apoptosis by chemotherapy. The CSCs niche is a dynamic supportive system with specific anatomic and functional features to modulate how the cells take part in the process of tumor growth and metabolism (Scadden, 2006).

Folkins et al. (2007) reported that instead of targeting the CSCs directly, attacking the vascular niche to collapse the resistance ability of tumor against the chemotherapy. In another case, Gulotta et al. (2007) suggested that in order to destroy the drug-resistant brain tumor, to attack the key member of CSCs niche-endothelial cells indirectly would be a promising strategy. The anti-inflammatory drugs (IFN-β) could also serve as potential components of CSC-focused therapies to disrupt the vascular niche of glioma CSCs (Williams et al., 2010).

Whereas neither antiangiogenic nor tumor cell cytotoxic effects alone are sufficient to reduce the CSCs fraction, therapies combining these effects are capable of selectively eliminating CSCs from the tumor. The possible reason for these observations is that antiangiogenic therapies disrupt a glioma CSCs vascular niche. The subsequent loss in communication between CSCs and their niche would elicit a reduction or loss of certain stem cell characteristics, which could include aspects of stem cell-associated drug resistance, and may result in increased proliferation rate and reduced DNA repair capacity in CSCs. Such changes would sensitize the CSCs to the cytotoxic effects of cyclophosphamide, therefore allowing combinatorial therapies to selectively eliminate CSCs.

### Inhibition of CSCs-Dependent Signaling Pathways

**FIGURE 2:**
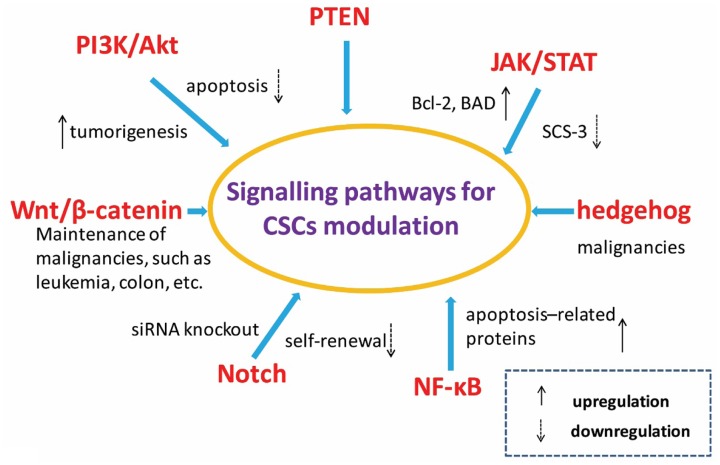
**Signal pathways related with cancer stem cells.** Pathways and elements involved in the control of selfrenewing and differentiation of cancer stem cells as well as normal stem cells include PI3K/Akt, JAK/STAT, Wnt/β-catenin, hedgehog, Notch, NF-κB, and so on.

The maintenance of stem cell nature partly depends on the regulation of relative signaling pathways. Several signaling pathways have been implicated in CSC chemoresistance in a variety of cancers. The pathways and signal molecules related to the control of CSCs self-renewal, differentiation and apoptosis include PI3K/Akt, PTEN, JAK/STAT, Wnt/β-catenin, hedgehog, Notch, and NF-κB etc. as shown in **Figure 2** (Gulotta et al., 2007; Baud and Karin, 2009; Burger and Peled, 2009; Chen et al., 2009; Guo et al., 2010; Lorenzo et al., 2010; Roy et al., 2010; Murphy and Stordal, 2011).

The activation of signal molecule Akt plays a critical role for cellular transformation and tumorigenesis. The PI3K/Akt signaling pathway regulated the growth of numerous cancers. Guo et al.’s (2010) study showed that Akt1 improved the resistance to apoptosis by increasing the expression level of Bcl-2 and the phosphorylation of the pro-apoptotic protein BAD. The tumor generation related signaling pathway JAK/STAT was activated at the same time. To control the suppressor of cytokine signaling-3 (SCS-3) is also viewed as an effective method to regulate tumor formation by the activating JAK/STAT signaling pathway (Pradhan et al., 2007). The Notch signaling pathways have also been shown to be involved in tumor development, metastatic initiation and self-renewal processes. The over expression of Notch3 could lead to the expansion of CSCs and chemoresistance enhancement in ovarian cancer. The sensitivity of CSC was obvious increased to platinum therapy after inhibiting the Notch signaling pathway via siRNA knockout. In addition, the well-known hedgehog and Wnt signaling pathways also play fundamental roles in maintaining CSC populations (Dontu et al., 2011).

### Targeting CSC-Dependent Markers

With the recent advances and growing knowledge on unique CSC biomarkers, tumor targeted therapy has shed new light to the cancer patients. The expressions of CSC surface markers, however, are heterogenous, which are different from patients with the same cancer and the same tumor in different progress stages. Thus, it’s critical to evaluate the CSC markers in the tumor of every stage to pick up the CSC relative treatment. Furthermore, the CSC surface marker proteins play important roles not only in cell isolation, identification and observation, but also in the applications of both preclinical diagnosis and clinical therapy (Visvader and Lindeman, 2008).

These markers include CD34, CD44, CD90, CD133, and EpCAM, etc. (Medema, 2013). The ligands or antibodies against tumor surface makers have been widely employed to enhance the specificity of therapeutic strategies. The anti-bodies of these CSCs biomarkers were developed and utilized in several drug delivery systems for tumor targeting and cancer therapy. Bourseau-Guilmain et al. (2011) prepared lipoprotein-like nanocapsules to deliver AC133 to effectively target CD133 positive CSCs. A CD44-specific tumor-targeting drug was carried by HA for the cancer therapy (Marhaba et al., 2008). An anti-CD90 antibody was also proved to be able to stop the bone marrow-derived multipotent stromal cells to differentiate into chondro-, osteogenic, or adipo-lineages (Gundlach et al., 2011).

### Interfering the CSCs Differentiation

Although the anticancer drugs for ideal targeting have being looked for decades, only few are successfully applied in clinical trial. One of them is all-trans retinoic acid (ATRA), Warrell et al. (1993) found that the differentiation of leukemic promyelocytes were blocked and failed to differentiate into mature granulocytes after treating the patients with ATRA. The rational theory of ATRA therapy is ATRA could drive the CSCs to differentiate from stem cell into non-CSCs (e.g., epithelial cells). This case indicated the therapy of interfering CSCs differentiation may be an effective way to treat other forms of cancers. Other agents such as phorbol myristate acetate (Carey et al., 1996), hexamethylamine bisacetamide (Wu et al., 1991), dimethylsulfoxide (Arcangeli et al., 1993) and vitamin D3 (Olsson et al., 1984) also were reported to have the similar function in CSCs differentiation therapy.

### Immunotherapy against CSCs

Challenges in CSCs targeting remain intractable issues need to be faced in a long term, while immunotherapy becomes a novel potential interest in the field of cancer therapy. Numerous researches have shown that CSCs can be eradicated by innate immune effector cells. The usage of tumor antigens to directly break the cancer patient’s immune system has been explored decades for cancer therapy (Pardoll, 2012). The strategies include: (a) using the specific peptides derived from tumor antigens for cancer vaccination; (b) stimulating the immune cells with dendritic-cells to fight against specific antigens; and (c) interfering the immune responses of anti-tumor via retardant.

The CSCs-secreted TGF-β that has additional immunosuppressive effects on other subclasses of T lymphocytes, was used to avoid being attacked by various cellular components of the immune system (Pardoll, 2012). Castriconi et al. (2009) indicated that the allogeneic and autologous-activated natural killer cells were capable of influencing the stem-like cells to lysis. Todaro et al. (2009) research demonstrated that γδ T lymphocytes can be used to kill human colon CSCs *in vitro*. Contag et al. (2010) reported that the oncolytic virus can be delivered into lymphoma cancer stem like cells as vehicles via cytokine-induced killer cells.

### Exploiting Metabolic Differences to Target CSCs

The behaviors of CSCs in the niche are affected by the metabolic products, the CSCs therefor can respond to the conditional environment. Because of the fresh discoveries in the Warburg effect, cancer metabolism has been coming back into spotlight the recent 10 years. This presents that cancer cells generate ATP through glycolysis (even under non-hypoxic conditions) rather than oxidative phosphorylation (Warburg et al., 1952). Another research reported that they set up a mouse model of lung CSCs and proved repressive functions of LKB1 on metastasis through regulation of NEDD9 and found that LKB1 mutation correlates with lung CSCs. These findings establish that LKB1 suppresses lung tumorigenesis through at least three independent mechanisms influencing tumor initiation, differentiation, and metastasis (Ji et al., 2007). With the development of novel therapeutics based on the continuous studies on signaling regulating metabolic pathways, the role of cell metabolism is shedding new light in cancer research. For instance, the metabolism of cancer cells can be changed via inhibiting the mTOR signaling by hypoxia (Wouters and Koritzinsky, 2008). Inhibiting the mevalonate metabolic pathway which plays vital role in breast CSCs generation by hydroxy-3-methylglutaryl CoA reductase blockers significantly break the CSCs growth process (Ginestier et al., 2012). These cases indicated that tuning the CSC metabolism could be considered as an effective way to interfere the CSCs activities by specific targeting and kill the CSC cells from the normal cells.

The CSCs extend our horizons of knowledge on cancer and provide a new approach to eradicate malignancies. It is proved that CSCs exist in various malignancies and play critical role in tumor initiation and development. While the conventional cancer therapy ways which are effective in various cancer treatments, have no more obvious effect in chemo- and radio-resistant cancer stem cells. Thus, a wide variety of CSCs targeting strategies are being investigated for the treatment of malignant tumors. What’s more, the unique characteristics of CSCs remain needed to be further explored to promote the understanding and utilization of CSCs. The strategies mentioned above are just a part of ways effectively used in CSCs targeting therapy. Although promising, the challenges of specificity and potential toxicities still need to be further tackled before the clinical trials. The drugs used for CSCs targeting based on their characteristics may have some adverse effects on the maintenance of normal cells or organs. Therefore, it will be critically important to develop new therapies that exclusively target CSCs and avoid targeting normal stem cells or cells in the future.

## Polymeric Drug-Loaded Nanoparticle Delivery System

Although the huge progress emerges in medical technique, the cancer therapy remains a challenging because of the low cure rate. The polymeric drug-loaded nanoparticles have been viewed as a novel promising strategy for cancer treatment because they not only can improve the drug pharmacokinetics but also further response to the permeation and retention (EPR) effect to enhance the accumulation of drugs at the site of the tumor during cancer treatment (Kapoor et al., 2015).

There are lots of factors should be included in an ideal design of polymeric nanoparticles system for drug delivery, including biocompatibility, particle size, drug loading rate, and pharmacokinetics, etc. (Vinogradov and Wei, 2012). The nanoparticles make themselves better cell affinity and easier uptake by the target cells through interactions ligands on the nanoparticles’ surface with receptors on the cell membranes. There are numerous advantages of nanoparticles using for anti-cancer drugs delivery in cancer treatment: (a) Increase the drug circulation half-lives: Polyethylene glycol, polyvinyl alcohol, polysaccharides, and surfactants such as poloxamer were used to decorate the surface of nanovehicles to circumvent the opsonization and the reticuloendothelial system. Cargos in these nanovehicles turned into decreased apparent clearance, extended apparent circulation half-life, as well as lessen apparent volume of distribution, eventually producing prolonged drug retention time in the circulation system. (b) Improve the drug aqueous solubility and stability: nanotechnologies are broadly drawing attention as favorable option for oral administration of drugs with poor oral bioavailability. Besides increasing drug uptake via cell-mediated active absorption (Florence, 2005), drug encapsulated nanoparticles could diminish transporter-mediated efflux (Ling et al., 2010). Additionally, drug loaded in nanoparticles protects the drug from degrading by harsh conditions in the GI system (Italia et al., 2007; Mittal and Kumar, 2009) and decreases hepatic first-pass metabolism (Anand et al., 2007; Mittal et al., 2007). Drug loaded in nanoparticles could safely go through the gastrointestinal tract and liver avoiding degradation/metabolism, which would result in reduction of the metabolic clearance and increasing of its absorption and oral bioavailability as well. (c) Control the drug release and specific targeting: some of the most amazing advantages of nanocarriers include their tunable payload release and the ability to specifically target their payload to diseased tissues and cells by modification of their surface chemistries, and finally their ability to respond to various internal and external stimuli for “triggered” release to achieve temporal and spatial control over the release of therapeutic payloads. (d) Enhance the drug bioavailability: nanotechnology is increasingly considered to be the technology of the future. Among the wide applications of nanotechnology is the use of nanoparticles for enhancing the bioavailability. Poorly water-soluble drug candidates encountered in drug discovery cause increasing problems of poor and variable bioavailability. It was suggested that nanoparticles could enhance the oral drug bioavailability probably because the special stability (thermodynamic and kinetic stability) facilitate the safe transport of drugs through the G1 tract and/or the mucoadhesivity of nanoparticles prolonging the residence time in the gut, and the P-gp inhibitors contributing to drug accumulation.

### Designs of Nanoparticles for Drug Delivery System

**FIGURE 3:**
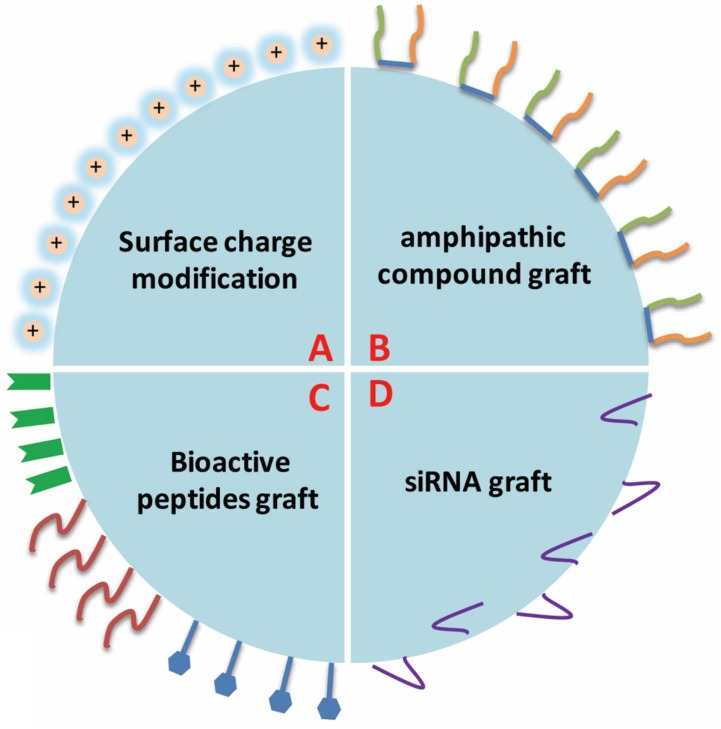
**The usual surface modifications of polymeric nanoparticles.** The usual modification includes surface charge modification, bioactive peptides graft, amphipathic compound graft, and siRNA, etc.

Although the traditional cancer therapy techniques are improving and various novel therapeutic strategies are being developed, polymeric nanoparticles are still viewed as the first option for drug delivery and also widely used in the research of other diseases due to their excellent physicochemical properties (Fonseca et al., 2013, 2014), such as particle size, surface charge, surface chemistry, hydrophobicity, degree of rigidity, and degradation speed. The polymeric nanoparticles also can be decorated on the surface to obtain the appropriate functions to fit the tumor environment or properties of drugs. The usual modification includes surface charge modification, bioactive peptides graft, amphipathic compound graft and siRNA (**Figure 3**).

Surface modification of polymeric nanoparticles has been proved to be useful in offering multi-functionality, and cannot only easily optimize the nanoparticles distribution in cells and tissues, but also promote cellular uptake or reduce cellular interactions. The modified polymeric nanoparticles can accumulate the loaded-drugs within the tumor tissues accurately and avoid damaging the nearby healthy tissues.

#### Particle Size

One of the most widely utilized mechanisms of drug delivery system for targeting is based on particle size. The enhanced EPR effect allows nanoparticles less than 500 nm to penetrate tumor vasculature and gather in the regions of tumor (Petros and Desimone, 2010). The larger nanoparticles would be prevented getting there because they are trapped in the lungs or removed out the body by the reticuloendothelial system (RES). The nanoparticles are usually modified to obtain neutral surface charge by coating hydrophilic molecules such as PEG to eliminate particle aggregation and avoid RES for the EPR effect (Torchilin, 2011).

#### Transferrin

Transferrin (Tf) is a glycoprotein used to improve targeting efficiency for the tumor cells which overexpress the Tf-receptor by grafting to nanoparticles made of poly(L-lysine) (Wagner et al., 1990), polyethyleneimine (Ogris et al., 2003), cyclodextrin (Bellocq et al., 2003), or polyamidoamine (Huang et al., 2007). Tf was reported to be conjugated onto the surface of PEI and PEI-PEG nanoparticles for plasmid delivery, which showed the clear improvement of tumor targeting and reduction on off-target transfection (Hildebrandt et al., 2003). Tf conjugated to cyclodextrin-PEG nanoparticles (CALAA-01) for the delivery of small interfering RNA (siRNA) to solid tumors is undergoing FDA Phase I clinical trials (Davis, 2009).

#### Epidermal Growth Factor Receptor

The epidermal growth factor receptor (EGFR), which considered as a signal of poor prognosis, overexpressed in approximately 30% of solid tumors during cancer therapy (Jones et al., 2006). The EGFR targeted polymeric nanoparticles were shown to improve tumor specific transfection compared to non-targeted nanoparticles (Kathrin et al., 2011).

#### RGD Peptide

The RGD peptide (arginine, glycine, aspartic acid) is reported to strongly target the αvβ3 integrin receptor which is selectively expressed in tumor vasculature to enhance the cellular uptake, and weakly bind to some other integrin receptors (Kunath et al., 2003). PBAE nanoparticles attached by RGD peptide for the targeted delivery of DNA to tumor cells was proved to improve transfection of cells overexpressing integrin receptors (Green et al., 2007). Both the RGD peptide and cyclic-RGD peptide conjugated to nanoparticles for targeted delivery of plasmids to tumor cells has also been shown to improve transfection *in vivo* for 24 h after IV administration in mice (Sakae et al., 2008).

#### Antibodies

Antibodies can be employed to specifically target a tumor that expresses a tumor specific antigen. PEI conjugated with HER2 antibodies for DNA delivery improved transfection against HER2^+^ breast cancer cell lines *in vitro* (Chiu et al., 2004). PEI/DNA nanoparticles conjugated with antibodies of prostate specific membrane antigen for targeting prostate cancer cells *in vivo* was reported to have a 20 times enhancement compared to non-targeted nanoparticles (Moffatt et al., 2006).

In additionally, Folate (Zhang et al., 2010), HA (Needham et al., 2012), and some specific carbohydrates have been also employed as targeting moieties for appropriate polymeric nanoparticle delivery to CSCs.

The increasing attentions on approaches of cancer treatments have raised the research interest of CSCs targeting via polymeric nanoparticle. The use of ligands conjugated to the surface of nanoparticles can allow the drugs enter CSCs and regulate lots of genes or protein expression, kill the CSCs eventually. To improve the transfection efficiency and the capacity of cellar uptake, the factors such as ligand density, surface charge and affinity have to be considered in the process of material design.

### Applications of Polymeric Nanoparticles for CSCs Targeting Therapy

Polymeric nanoparticles have been regarded as the most efficient carriers for drug delivery due to their excellent pharmacokinetic properties such as drug loading, drug release, structure stability and nanoparticles degradation (Alexis et al., 2010; Pérez-Herrero and Fernández-Medarde, 2015). The recent research hotspot of polymers utilized for drug loaded nanoparticles include poly (D, L-lactic-co-glycolic acid) (PLGA), polylactic acid (PLA), poly (ethylene glycol) (PEG), chitosan (CS), and hyaluronic acid (HA), etc.

#### Poly (lactide-co-glycolide)

Poly (lactide-co-glycolide) (PLGA) is one of the few US FDA approved polymers for clinical applications due to its outstanding properties such as biodegradability and biocompatibility (Chaubal, 2002; Astete and Sabliov, 2006). So far, it’s the most widely used synthetic polymers in drug-loaded nanoparticles development for cancer therapy (Biondi et al., 2008). Ni et al. (2015) also reported that the CD133 grafted PLGA nanoparticles loaded with salinomycin were capable to target the CD133^+^ osteosarcoma CSCs.

To kill both the normal cancer cells and CSCs, salinomycin (SLM) and paclitaxel (PTX) were embedded by PGLA for CD44 targeted chemotherapy with emulsion solvent diffusion method. This combinational therapy demonstrated the synergistic effects of dramatically reducing the CD44^+^ cells (Muntimadugu et al., 2016). In order to eliminate drug resistance and relapse of breast CSCs, Swaminathan et al. (2013) prepared PLGA nanoparticles to target CD133 via conjugating an anti-CD133 monoclonal antibody. The result showed that the breast CSCs population was significantly reduced and the therapeutic efficacy was obviously enhanced. To further actively target CSCs to reduce their drug resistance and stabilizing agent, HA and PF127 were conjugated onto PLGA to get the smart nanoparticles for cancer therapy. These nanoparticles were synthesized using the double-emulsion approach and allowing for acidic pH-triggered drug release and thermal responsiveness at the same time (Wang et al., 2015).

#### Hyaluronic Acid

Hyaluronic acid (HA) is an anionic, non-sulfated glycosaminoglycan distributed in connective, epithelial and neural tissues. HA has been widely studied in cancer treatment by conjugating different drugs due to its outstanding characteristics including biocompatibility, biodegradability, and non-immunogenicity (Luo et al., 2000; Prestwich, 2011). Many drug-resistant cancer cells and CSCs were reported to enhance the expression levels of some biomarker receptors. These markers conjugated onto HA can use to target CSCs for the cancer therapy. For instance, cholesterol-modified HA (CHA) conjugated with selected drugs that are highly active against CSCs via biodegradable covalent linkage can be used to deliver the drug efficiently for drug-resistant cancer. Banzato et al. (2008) developed a paclitaxel-hyaluronan bioconjugate (ONCOFID^TM^-P, 20% drug content) to target human ovarian cancer IGROV-1 and OVCAR-3 xenografts via the intraperitoneal administration. The HA was also structurally functionalized for siRNAs encapsulation to transfect siRNAs into cancer cells that overexpress CD44 receptors. Several HA based drug-loaded nanoparticles delivery systems have been delivered into the cancer cells overexpressed the CD44 receptors, and some decorated HA derivatives can effectively silence the related gene activity *in vitro* and *in vivo* (Choi et al., 2009).

However, there is a defect of HA-based drug delivery nanoparticles in cancer therapy application. The delivery system preferentially accumulates in the liver after systemic administration. To address this issue, poly (ethylene glycol) is employed to conjugate onto HA. The result indicated that the PEG-modified HA-nanoparticles were significantly reduced in the liver, and lengthened the times stay in blood circulation, eventually enhanced the veracity of CSCs targeting (Choi et al., 2011).

#### Poly (ethylene glycol)

Poly (ethylene glycol) (PEG) is a hydrophilic polymer widespread use in the field of biomedicine due to its excellent water solubility, chain mobility, non-toxicity, and non-immunogenicity. In order to solve the low water solubility and unstable properties of bortezomib in clinical application, the amphiphilic copolymer poly (ethylene glycol)-block-poly(d,l-lactide) (PEG-b-PLA) nanoparticles were engineered. Their study demonstrated that the bortezomib loaded in PEG-b-PLA nanoparticles can enter into the targeted CSCs, and induce the CSCs apoptosis and death. This DDS was proved to keep the drugs in CSCs for a long time and accumulate the drugs in lesion tissue, and eventually inhibit the proliferation of CSCs, promote the therapeutic efficacy in breast cancer therapy (Shen et al., 2015). To lower the toxicity and improve the drug release, the diblock copolymer nanoparticles of poly (ethylene glycol) (PEG) and functional polycarbonates (PAC) were fabricated via metal-free organocatalytic ring-opening polymerization to provide the narrow size distribution and high loading capacity for anticancer drugs. Doxorubicin (DOX)-loaded PEG/PAC nanoparticles showed higher antitumor efficacy and decreased toxicity than free DOX *in vivo* (Ke et al., 2014). To increase the solubility, the potent vascular-disrupting agent was bonded to a PEG-based polymer to achieve self-assemble in the aqueous solution. DOX then was loaded into these nanoparticle carriers to improve the cytotoxicity and loading capacity. The *in vivo* study on MCF-7 tumor bearing nude mice demonstrated that the antitumor efficiency was significantly improved (Vinogradov and Wei, 2012). To make the PEG nanoparticles intelligent, Liu et al. (2013) developed the gelatinases-stimuli poly (ethylene glycol)-peptide-poly(𝜀-caprolactone) drugs delivery system. The nanoparticles were developed to investigate the targeting effects on CSCs and non-stem cancer cells by carrying both miR-200c and docetaxel (DOC). The results demonstrated that the tumors growth speed was decreased after the miR-200c/DOC nanoparticles were put into the nude mice. The PEG-based gelatinases-response drug-loaded nanoparticles may also suggest that the loading of both drugs and siRNA can target not only CSCs but also non-stem cancer cells at the same time.

#### Polylactic Acid

polylactic acid (PLA) is FDA approved biodegradable polymer and fumaric acid existed in the Kreb cycle. It can be totally excreted through metabolism after degradation. Yang et al. (2015) established lung cancer stem-like cells (CSLCs)-targeting encapsulated docetaxel PLA nanoparticles for anti-metastatic therapy. The *in vitro* and *in vivo* experimental results confirmed the outstanding anti-metastatic performance of CSLC-targeting DTX nanoparticle. Liang et al. (2015) also have the similar study, they fabricated the PLA nanoparticles loading DTX based on the emulsion solvent evaporation method. Their study indicated that the tumor growth inhibition and anti-metastatic efficacy in the SCLC targeting drug delivery system for cancer treatment. Pandey et al. (2015) developed PLA nanoparticles to deliver quercetin (Qt) to inhibit the human breast cancer cells. Results showed that the PLA-Qt nanoparticles displayed excellent sustained release kinetics and can be used as a novel vehicle for the cancer treatment. In another case, the cellulose, that was reported to protect drugs and deliver them to the target site, was grafted on poly (L-lactic acid) (PLLA) to load betulinic acid for anti-cancer therapy. Results demonstrated that the experimental effect in the CEg-PLLA/BA nanoparticles group showed not only fewer side effects but also better efficacy in tumor proliferation suppression than that of BA alone (Dai et al., 2014). To regulate the signal molecules related to breast CSCs, the MPEG-b-PLA nanoparticles were developed to deliver the small molecules (Ahmed and Discher, 2004; Blanco et al., 2007). Low-dose of DAC encapsulation can decrease the number of CSCs in mammospheres of MDA-MB-231 cells and rectify their sensitivity to DOX. While systemic delivery of DAC cannot only obviously decrease the expression level of DNMT1 and DNMT3b in a MB-MDA-231 xenograft murine model, but also up-regulate the caspase-9 expression to enhance the sensitivity of CSCs to DOX. The controllable MPEG-b-PLA nanoparticles loading capacity offers a promising strategy for breast cancer therapy (Li et al., 2014).

#### Chitosan

Chitosan, derived from chitin, is a positive biocompatible and biodegradable natural polysaccharide. It is widely used in the field of biomedicine including drug and gene delivery, tissue engineering, wound healing, and antimicrobial (Şenel and Mcclure, 2004; Riva et al., 2011). The chitosan nanoparticles were developed for targeting the overexpressed CD44 receptors on CSLCs. The nanoparticles around 20 nm were delivered into the tumor to actively target and be internalized in CD44^+^ CSLCs by EPR effect. The drugs in nanoparticles then would release out and diffuse into the cytosol, eventually accumulate in the nuclei to produce cytotoxicity to kill CSLCs eventually (Chauhan et al., 2012; Sykes et al., 2014). To lower the toxicity of platinate, Nascimento et al. (2015) developed the chitosan-based polymeric nanoparticles to encapsulate hydrophobic drugs by grafting PEG and peptides. Their study reported that the modified platinate encapsulated into chitosan-based nanoparticles can obviously decreased the toxicity in cells. The chitosan also can be used as cell recognition. A doxorubicin-encapsulated polymeric nanoparticles decorated with chitosan on the surface were developed to specifically target the CD44 receptors of the cancer treatment. This nanoparticle system released the doxorubicin in acidic environments localized in the cellular endosomes/lysosomes to achieve the goal of killing the CSCs (Rao et al., 2015).

In summary, a large number of polymeric nanoparticles with different physicochemical characteristics for multiple drugs loading have been developed to control the drugs release via responding internal or external stimuli to significantly improve therapeutic efficacy by CSCs targeting.

## Conclusion and Prospects

Cancer is one of the most complicated diseases. Although the drug-loaded nanoparticles have improved preclinical and clinical outcomes for cancer treatment, the therapeutic efficacy is still limited due to the genetic diversity and the biological complexity of tumor. Therefore, modifying the chemical or physical characteristics of polymeric nanoparticles to meet the need of different drugs with different physicochemical characteristics is an attractive strategy for clinical CSCs targeting.

In this review, we introduce the ways of CSCs inhibition based on the CSCs profiles. Then we enumerate several representative decorated polymeric nanoparticles for drugs delivery in the applications of cancer therapy.

More and more studies have confirmed the CSCs is critical factor for tumor resilience, resisting to radiotherapy, and were accused for tumor Resurrection. As that knowledge about CSC advanced, cutting-edge ideas and technologies have kept coming forth in order to eliminate malignancies. Several kinds of carcinomas may harbor CSCs with mesenchymal properties, and self-renewal and multi-differentiation properties, while it’s hard to find an exclusive selection marker to isolate and target the corresponding CSCs. Fractions of CSCs could be masked by a tumor with heterogeneousness, that indicate targeting treatment for CSC isn’t a simple task. Therefore, many different targeted therapies for systemic treatment for CSCs have been put forward recently. The studies mainly focused on inhibition of the VEGF and mTOR pathways and targeted on markers (such as CD105 and CXCR4) of CSCs. Although various fresh trials have been carried out in clinic, there are still observable restrictions occur in getting to CSCs *in vivo* due to the unreachability of the deep area of tumor or because of the tumor milieu, these restrictions could be overpowered by nanotechnological strategies. Versatile CSC-targeting approaches together with newly discovered pathway and cytotoxic agents could contribute to tackle those difficulties in CSCs remedy. Accumulating knowledge on CSCs will lead us to comprehensively understand the responses of CSCs to various treatments, different phases of disease, and individual patient. Finally, all of these finding would bring about more patient-tailored and targeted treatments. Sensibly built-up nano-therapy for CSCs explores new routes for clinical study and brings in new faith in practical use.

In the recent years, the hot research interest mainly focusses on design of novel polymeric nanoparticle-based therapies for CSC targeting in cancer treatment. Polymeric nanoparticles can be expediently modified with the cell-recognizable moieties on their surfaces, and designed in appropriate size and structure for pharmaceutical applications. The polymeric nanoparticle-based approaches have increased the drug safety, controllable drug release, and targeting efficacy, and lowered the immunogenicity and tumorigenesis.

Novel therapeutic strategies for combination therapy and stimuli-activated drug delivery are also the representative of new therapeutic trends. For instance, to deliver two or more drugs simultaneously would improve different tumor growth-related signaling pathways targeting, leading to a synergistic therapy effect.

However, these approaches are still in the early stages of development and have many theoretical and technical limitations, issues of dose-related toxicity and side effects are also needed to be addressed. Furthermore, the innovative *in vitro* evaluation system has to be improved to accurately predict the *in vivo* pharmacokinetics of drugs in humans. In spite of small animal models have been used to evaluate the therapeutic effect, it is far away from a perfect predictor of the drug efficacy in humans.

The revolutionary developments of novel drugs and new drug delivery systems are urgently needed, and the *in vitro* models and *in vivo* pharmacokinetic profiles also need to be improved to enhance the CSCs targeting for the cancer therapeutic effect.

## Author Contributions

BL summarized the literature, wrote the manuscript and drew the figures. QL wrote the manuscript and revised the manuscript. HD and JM supervised all the works and provided critical comments.

## Conflict of Interest Statement

The authors declare that the research was conducted in the absence of any commercial or financial relationships that could be construed as a potential conflict of interest.
